# Sensitivity and specificity of vessel wall MRI sequences to diagnose central nervous system angiitis

**DOI:** 10.3389/fstro.2022.973517

**Published:** 2022-08-18

**Authors:** Lorenzo Ferlini, Noemie Ligot, Arab Rana, Lise Jodaitis, Niloufar Sadeghi, Virginie Destrebecq, Gilles Naeije

**Affiliations:** ^1^Department of Neurology, Erasme Hospital, Université Libre de Bruxelles, Brussels, Belgium; ^2^Department of Neuroradiology, Erasme Hospital, Université Libre de Bruxelles, Brussels, Belgium

**Keywords:** MRI, vessel wall imaging, CNS–central nervous system, vasculitis, intracranial stenosis

## Abstract

Magnetic resonance imaging (MRI) with intracranial vessel wall (IVW) sequences is able to directly characterize disease processes affecting the VW increasing the accuracy of intracranial vasculopathies differential diagnosis. Nevertheless, data concerning the specificity and sensitivity of this technic for diagnosis of angiitis of the central nervous system (ACNS) are scant. We aimed at quantifying the IVW abnormalities in a cohort of primary and secondary ACNS and assessing the specificity of ACNS-associated IVW MRI abnormalities. We retrospectively included 36 patients with a diagnosis of ACNS with IVW imaging and we compared IVW MRI abnormalities with those of fifty successive patients admitted at the stroke unit for acute neurological deficit (stroke-unit-group). Since an ACNS was retained as cause of the acute neurological deficit in two patients in the stroke-unit-group, they were finally included in the ACNS group. In our cohort, headache and cognitive disorders were present in 29 and 55% of cases respectively, seizure were rare (*n* = 5, 13%). CSF analysis were abnormal in 29 patients (76%). MRI showed lesion with restricted diffusion in 82% of patients (*n* = 31). In 71% of our ACNS population (*n* = 27) multifocal vessel abnormalities were described in angiographic MRI sequences. Digital subtraction angiography (DSA) was performed in 14 patients and it was suggestive of an ACNS in 11 (79%). Brain-meningeal biopsy was performed in two cases (5%). The typical ACNS-associated IVW abnormalities, i.e., the multifocal concentric VW enhancement with wall thickening, was found in the 95% of ACNS patients (*n* = 36) whereas it was reported in 4% (*n* = 2) of the stroke-unit-group (specificity and sensitivity of concentric VW enhancement for ACNS diagnosis of 95% and 94%, respectively). IVW enhancement co-localized with multifocal angiographic stenosis in ACNS patients. The clinical, laboratory and imaging findings were comparable to those of previously described ACNS cohorts in the literature, and particularly to those of DSA-diagnosed patients. Our results suggest that concentric VW enhancement could efficaciously identify patients affected by medium-sized vessels CNS vasculitis with a specificity of 95% and sensitivity of 94%. Further studies with larger samples are necessary to confirm our findings.

## Introduction

Intracranial stenosis of the cerebral arteries is a common cause of ischemic stroke (Hurford et al., 2020) and the risk of stroke recurrence remains high despite aggressive medical management (Turan et al., 2017). Invasive luminal imaging techniques, i.e., the digital subtraction angiography (DSA), has traditionally been considered the gold standard to evaluate intracranial vascular diseases (Alexander et al., 2016). However, since many cerebral vasculopathies can have similar luminal imaging appearances (i.e., beaded appearance of the arteries with variable degrees of stenosis, ectasia, and occlusion), the ability of this technique to distinguish between different etiologies of vessel abnormalities is low (Alexander et al., 2016). In case of suspicion of angiitis of the CNS (ACNS), DSA holds a variable sensitivity (from 27 to 90%) and its specificity can be as low as 30% (Duna and Calabrese, 1995; Alexander et al., 2016; Singhal et al., 2016). Since disease morbidity and mortality can be substantially reduced by aggressive immunosuppressive treatment in case of ACNS [from 17 (Salvarani et al., 2015a) to 6% (de Boysson et al., 2014) if steroids are associated to cyclophosphamide], other invasive procedures, such as the brain meningeal biopsy (BMB), are sometimes considered to attest the diagnosis and avoid useless potentially hazardous therapy. However, serious post-operative complications rate of BMB is about 4%, and, in any case, it presents a limited sensitivity (50–74%) due to the heterogenous and segmental vasculitic vessel damage (Torres et al., 2016) and subtype (Schuster et al., 2017). Magnetic resonance imaging with intracranial vessel wall sequences (IVW) has witnessed a growing interest as recent studies showed an overall sensitivity approaching that of DSA for ACNS diagnosis (Boulouis et al., 2017; Destrebecq et al., 2020). Moreover, IVW can help differentiate vasculitis from other intracranial vasculopathies, namely intracranial atherosclerotic disease (ICAD), reversible vasoconstriction syndrome (RVCS) or intracranial arterial dissection. IVW abnormalities in vasculitis are characterized by a multifocal or diffuse homogenous concentric enhancement of the vessel wall that are usually thickened (Kanduumlker et al., 2008; Obusez et al., 2014). On the contrary, in ICAD the vessel wall enhancement is typically eccentric while in RVCS the vessel involvement is circumferential but no to mild enhancement has been reported (Mossa-Basha et al., 2015; Alexander et al., 2016). However, data on the specificity and sensitivity of IVW for ACNS diagnosis are scant. Building upon our cohort of primary angiitis of the CNS (PACNS) (Destrebecq et al., 2020) and secondary ACNS, we aim at (i) quantify the IVW abnormalities in ACNS on a larger cohort and (ii) assess the specificity of ACNS-associated IVW MRI abnormalities.

## Materials and methods

### Study design and population

We retrospectively considered for inclusion all patients admitted in the stroke unit of the Erasme Hospital (Université Libre de Bruxelles, Belgium) from January 2016 to January 2022 for acute neurological deficits for whom a diagnosis of vasculitis with a CNS involvement was retained after a thorough work-up to exclude, in addition, alternative stroke etiologies. The work-up consisted of a brain MRI with parenchymal and vascular assessment (including MRA and IVW sequences), a complete cardiovascular screening (including transthoracic and transesophageal echocardiography, carotid ultrasonography, at least 24-h Holter electrocardiogram recording), an immunologic and infection assessment, as described in Boulouis et al. (2017), a whole-body [18F]FDG PET to seek for malignancy and a lumbar punction. The diagnosis of ACNS was retained if luminal imaging (MRA or DSA) displayed the typical multifocal intracranial vessel stenosis or a cerebral biopsy showed signs of vasculitis. If luminal imaging were non-contributory (i.e., showed unifocal stenosis), the diagnostic of ACNS may be retained if clinical history, parenchymal MRI and CSF analysis were suggestive of a vasculitic process and the thorough work-up did not provided a more likely etiology to the neurologic deficit. Results from IVW sequences were not considered for patient inclusion. In case secondary causes of CNS vasculitis were ruled out, a diagnosis of PACNS was retained. Vasculitis as a manifestation of bacterial and/or fungal infection were excluded, as time course and imaging differ significantly from the included conditions.

The compute accuracy of the IVW sequences to diagnose a CNS vasculitis was tested comparing the results of MRI vessel images in vasculitis patients with those of 50 consecutive patients admitted in the stoke unit for an acute neurological deficit (named, henceforth, the stroke-unit-group). The study was approved by the Ethics Committee of Erasme Hospital (reference P2020/736), which waived the need for an informed consent for the medical records review due to its retrospective design and for the prospective part of this work since VW imaging are already included in the standard imaging protocol of our institution in case of acute neurological deficit.

### Data collection

We collected demographic data, such as age, ethnicity, gender, medical history, and cardiovascular risks factors. Neurological symptoms at hospitalization in the stroke unit, as well as the results of laboratory investigations, including CSF fluid analysis when available, treatment and outcomes were recorded too. In particular, CSF analysis were deemed abnormal if leucocyte count exceeded 5 cells/mL, and/or total protein level >0.45 mg/mL, and/or intrathecal oligoclonal bands were present (Destrebecq et al., 2020). Clinical outcome was defined as either “stable/remission” or “progression” according to Salvarani et al. (2015b) and Patzig et al. (2022). Brain MRI protocol has been previously described (Destrebecq et al., 2020). Briefly, a 3T MRI scanner (Achieva, Ingenia; Philips Healthcare, Best, The Netherlands) was employed. Beyond standard sequences such as fluid attenuated inversion recovery (FLAIR), diffusion weighted Imaging (DWI), T1, T2, T2^*^, 3D time of flight (TOF) angiography and a 3D-T1 contrast-enhanced MRA, our protocol included a 3D turbo-spin-echo isotropic T1-weighted imaging with a black blood prepulse, before and after gadolinium administration. The presence of ischemic infarcts, hemorrhage and markers of small vessels disease (i.e., matter hyperintensities, cerebral microbleeds, lacunes, and dilatation of perivascular spaces) were collected. We systematically sought for the presence of vessels abnormalities (including stenosis and occlusions), the patterns of vessel wall contrast enhancement (either concentric or eccentric) and their localization (defined as proximal if vessels up to the second division branches were involved, and as distal for involvement of further division branches). The results of follow up IVW sequences was dichotomized in “stable/remission” if IVW abnormalities remained stable or improved and “progression” if they worsened.

### Statistical analysis

Statistical analysis were performed using BM SPSS^®^ Statistics for Windows version 25 software (IBM, Armonk, NY, USA). *P* < 0.05 was considered to indicate a statistically significant difference. Categorical variables were expressed as a count (percentage), whereas continuous data were presented as a median and interquartile range (IQR). Mann Whitney, Fisher's exact and χ^2^ tests were used to analyse differences in variables between ACNS and stroke-unit-group, as appropriate. The linear step-up procedure introduced by Benjamini and Hochberg was applied for controlling the false discovery rate (Benjamini and Hochberg, 1995). Specificity and sensitivity were calculated and defined as in the literature (Trevethan, 2017).

## Results

### ACNS cohort

**Figure 1:**
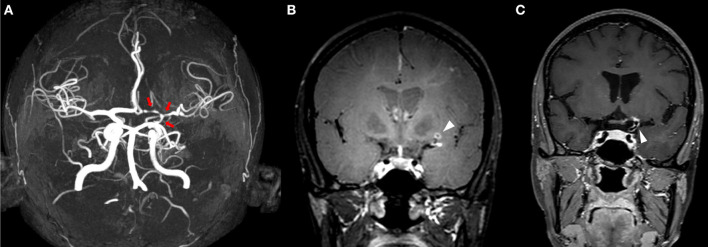
MRI of a patient presenting with a secondary ACNS in the context of systemic lupus erythematosus. Time of flight-MRA showing stenotic lesions in the terminal part of internal left carotid, middle and anterior left cerebral arteries [**(A)** red arrows]. IVW-MRI showing multiples areas of concentric contrast enhancement in the terminal part of internal carotid and in the middle artery in the left side [**(B,C)** arrowheads].

**Figure 2:**
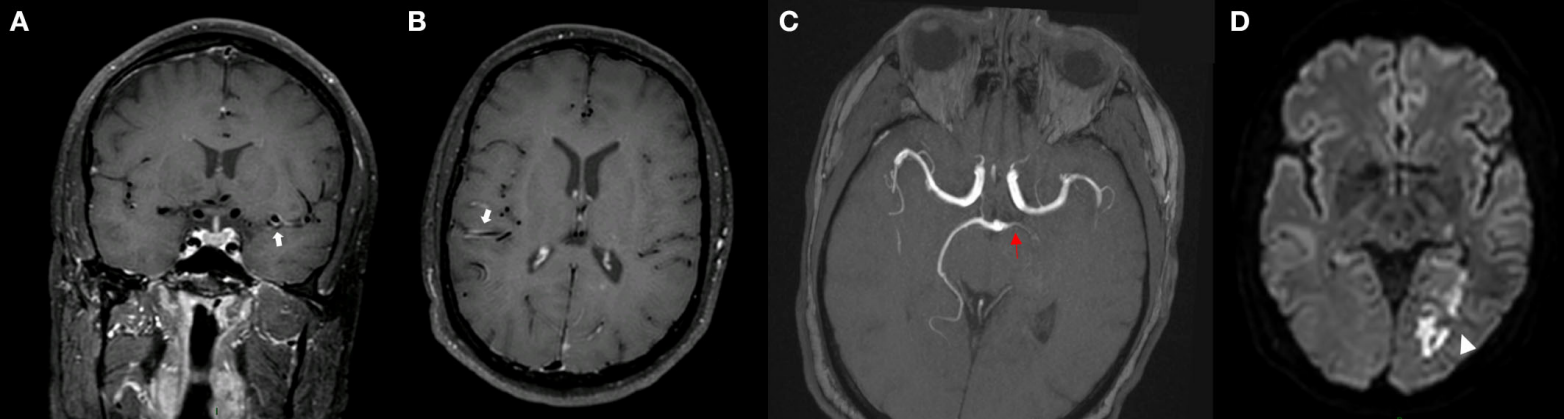
MRI of a patient presenting with PACNS. IVW-MRI showing multiples areas of concentric contrast enhancement in the proximal left and distal right the middle cerebral arteries [**(A,B)** arrows]. Time of flight-MRA showing a stenotic lesion of the left posterior cerebral artery associated with an acute infarct in the left occipital lobe [**(C)** red arrow, & **(D)** arrowhead].

Thirty-six patients met inclusion criteria for ACNS. Two more patients were identified among the 50 non-selected stroke patients and included in the cohort (*n* = 38, Table 1). After a through work-up, a secondary vasculitis was deemed likely in fourteen cases (37%, Figure 1). Among the most frequent etiologies, four patients presented an infectious related process (29%, three secondary to an HIV and one to a HCV infection), in four, a Horton arteritis was diagnosed (29%) and a paraneoplastic process was retained in three other patients (21%). The remaining 24 (63%), in whom the work-up was negative, were considered as PACNS (Figure 2). Clinically, all patients presented with a focal neurological deficit, essentially a motor weakness (66%). Language disturbances were approximately as frequent as vertigo or visual deficit (42, 39, and 38%, respectively). Focal deficits were often associated with cognitive disorder (55%) and headache (29%), whereas seizure were rarer (13%). Cardiovascular risk factors, such as arterial hypertension, diabetes and dyslipidaemia, were present in relevant percentage of our population (84, 50 and 47%). CSF analysis were performed in 37 patients and the results were abnormal in 29 patients (76%). In parenchymal MRI, DWI sequences showed restricted diffusion in 31 patients (82%). T2^*^ sequences showed the presence of microbleeds or sequelae of parenchymal haemorrhagic lesions in 32% of patients. In only one case, there were signs of convexity subarachnoid hemorrhage. We recorded only one PACNS with a pseudotumoral presentation, characterized by a parenchymal, contrast enhancing, T2-hyperitense expanding lesion with perilesional oedema and haemorrhagic foci in the temporo-parietal left region. We did not note any other intraparenchymal nor meningeal contrast enhancement. Luminal imaging time of flight (TOF)-MRA showed multiple vessel stenosis and/or occlusions in 71% of patients, always interesting the intracranial vessel(s) responsible for the clinical symptoms too. IVW sequences showed a multifocal concentric enhancement and vessel wall thickening in 95% of patients, including the symptomatic stenotic vessel(s) but also other vascular territories without symptomatic lesions. A DSA was performed in 14 patients and it was deemed negative in three cases (21%). When positive, DSA showed intraluminal vessels anomalies, namely multifocal stenosis and/or distal irregularities, which were consistent with IVW imaging abnormalities. In two cases (5%), included the one with a pseudotumoral presentation, BMB was necessary to establish the diagnosis. In 27 (71%) patients, an immunomodulatory therapy was initiated. In the 30% of cases, patients received IV methylprednisolone pulses alone, whereas corticosteroids pulses were associated with immunosuppressant drugs (cyclophosphamide *n* = 13, rituximab *n* = 2, methotrexate and azathioprine *n* = 1 each) in the 63% of patients. One patient with a secondary ACNS received Mycophenolic acid in monotherapy. Five patients received only a treatment for the supposed cause of the ACNS (3 with a paraneoplastic and 2 with an infectious origin) and seven patients were lost to follow-up. Therapy allowed a clinical stabilization/remission of the pathology in 19 patients (61%) whereas 12 (39%) progressed. In 22 treated patients, data about repeated IVW sequences were available. Among stable/remission cases, the IVW contrast enhancement improved or remained stable in 10 (77%) whereas it progressed in three (23%). Among those with clinical progression, the IVW contrast enhancement improved or remained stable in three (34%) whereas it progressed in six (66%). The frequency of relapses was statistically higher in patients presenting with an IVW enhancement progression than in those with stable or improved IVW sequences (23 vs. 66%, *P* = 0.04).

**Table 1:** Clinical, biological and radiological characteristics of ACNS patients.

**Characteristics**		***ACNS (n =* 38)**	
		**n median**	**(%) [IQR]**
Age		60	[52.3–68]
Female		13	34%
CV risk factors			
	Hypertension	32	84%
	Hypercholesterolemia	19	50%
	Diabetes mellitus	18	47%
	Atrial fibrillation	3	8%
Clinical symptoms at presentation			
	Focal deficit	38	100%
	Headache	11	29%
	Cognitive disorders	21	55%
	Seizures	5	13%
PACNS		24	63%
Secondary		14	37%
	Infection related	4	29%
	Horton	4	29%
	Paraneoplastic	3	21%
	Other	3	21%
MRI & MRA			
	Focal vessel stenosis	4	11%
	Multiple vessel stenosis	27	71%
	Acute deep infarction	31	82%
	Multiples infarction	25	66%
	White matter lesions	33	87%
	ICH/MB	12	32%
IVW contrast enhancement			
	Multifocal	36	95%
	Proximal arteries	34	89%
	Distal arteries	28	74%
DSA		14	37%
Biopsy		2	5%
CSF analysis			
	Abnormal	29	76%
	> 5 white blood cells	8	21%
	> 45 mg/dL protein	23	61%
	Oligo clonal bands	10	26%
Treatment			
	Steroids alone	8	21%
	Steroids + immunosuppression	19	50%
Stable disease or remission		19	61%
	& IVW improvement	10	77%
	& IVW worsening	3	23%
Relapsing disease		12	39%
	& IVW improvement	3	36%
	& IVW worsening	6	64%

*MRI, magnetic resonance imaging; MRA, magnetic resonance angiography; ICH, intracerebral hemorrhage; MB, micro-bleeds; IVW, intracranial vessel imaging; DSA, digital subtraction angiography; CSF, cerebral spinal fluid*.

### Stroke-unit-group

**Figure 3:**
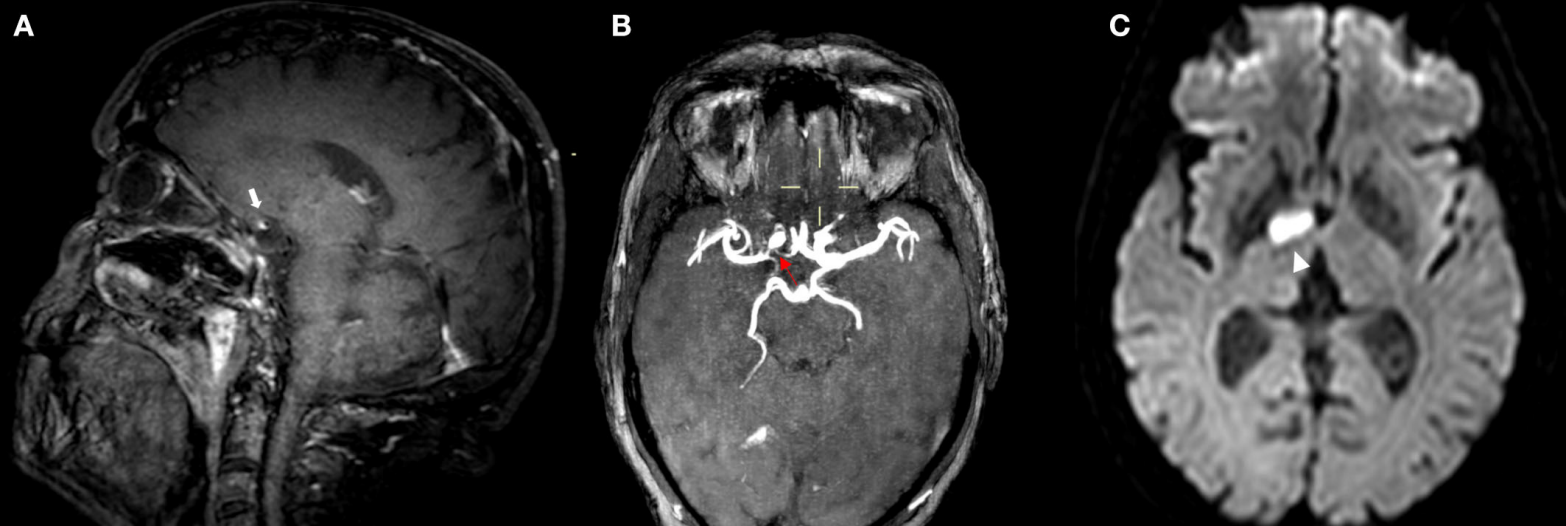
MRI of a patient presenting with ICAD. IVW-MRI showing eccentric contrast enhancement in the junction between right internal carotid and middle cerebral [**(A)** white arrow] which correspond to a stenosis in the same vessel segment in the time of flight-MRA sequence [**(B)** red arrow]. Patient presented with an acute infarction in the capsule-lenticular area [**(C)** arrowhead].

Clinical and radiological characteristics of our unit-group are resumed in Table 2. Our population presented hypertension and hypercholesterolemia in the 68 and 74% of cases, respectively, while diabetes and atrial fibrillation were less frequent (28 and 14%, respectively). Twelve patients (25%) presented headache at admission, while cognitive disorders or seizures were reported in a minority of cases. Thirty-two patients presented acute infarction lesions (64%) at diffusion MRI sequences. White matters lesion were present in 38% of patients while intracerebral hemorrhage or micro-bleeds were rarer (10% of cases). Twelve patients (25%) presented a VW contrast enhancement in the IVW imaging, seven unifocal and five multifocal. Among patients presenting with focal IVW abnormalities, five had an eccentric enhancement in the contest of an atherosclerotic stenosis (Figure 3), one in the context of a dissection and one of an aneurysm. In three patients, a multifocal eccentric enhancement was reported. In two cases, an intracranial atherosclerotic disease was diagnosed. In one case, the infarction origin remained uncertain but complete work-up did not provided evidence to support the diagnosis of ACNS. In two patients, the enhancement was concentric and multifocal. For one, the diagnosis of ACNS was deemed very likely but the work-up was incomplete since the patient requested discharge against medical advice. A patient presented multiples vessels stenosis and both concentric and eccentric vessel wall enhancements. A diagnosis of intracranial atherosclerotic disease was finally retained.

**Table 2:** Comparison between clinical and radiological characteristics in stroke-unit-group and ACNS patients.

**Parameters**		**Stroke-unit-group (*****n*** = **48)**		**ACNS (*****n*** = **38)**		***p***
		**n median**	**(%) [IQR]**	**n median**	**(%) [IQR]**	
Age		64	[54.8–75.5]	60	[52.3–68]	0.14
Female		24	50%	13	34%	0.16
CV risk factors
	Hypertension	33	69%	32	84%	0.14
	Hypercholesterolemia	37	77%	19	50%	**0.03**
	Diabetes mellitus	14	29%	18	47%	0.13
	Atrial fibrillation	7	15%	3	8%	0.52
Clinical symptoms at presentation
	Focal deficit	48	100%	38	100%	NA
	Headache	11	23%	11	29%	0.52
	Cognitive disorders	3	6%	21	55%	**0.004**
	Seizures	1	2%	5	13%	0.13
MRI & MRA
	Acute infarction	31	65%	31	82%	0.13
	Multiple vessel stenosis	17	35%	27	71%	**0.03**
	White matter lesions	17	35%	33	87%	**0.004**
	ICH/MB	4	8%	13	34%	**0.02**
IVW contrast enhacement		12	25%	36	95%	**0.004**
	Multifocal concentric	2	4%	36	95%	**0.004**
	Multifocal eccentric	4	8%	0	0%	0.15
	Unifocal	7	15%	0	0%	**0.03**
Stroke origine (TOAST)
	I. Large artery atherosclerosis	15	31%	0	0%	NA
	II. Cardioembolism	8	17%	0	0%	
	III. SVD	3	6%	0	0%	
	IV. Other etiology	2	4%	38	100%	
	V. unknown	8	17%	0	0%	
	> 2 etiologies	6	13%	0	0%	
Other diagnosis than stroke or TIA		6	13%	NA	NA	NA

*ACNS, angiitis of the central nervous system; MRI, magnetic resonance imaging; MRA, magnetic resonance angiography; ICH, intracerebral hemorrhage; MB, micro-bleeds; IVW, intracranial vessel imaging; SVD, small vessel disease; NA, not applicable; TIA, transient ischemic attack. Data are presented as median and range or number and percentage. Mann Whitney, Fisher's exact and χ^2^ tests were used to analyze differences in variables between groups, as appropriate. P <0.05 was considered statistically significant and marked in bold in the table after correcting for false discovery rate*.

### Comparison between ACNS and stroke-unit-group

Differences between ACNS and the stroke-unit-group are resumed in Table 2. Among cardiovascular risk factors, ACNS patients tended to present more frequently with arterial hypertension and diabetes and less frequently with dyslipidaemia, although only dyslipidaemia was statistically significant (*P* = 0.03). Cognitive disorders were more frequent in the ACNS patients (55 vs. 6%, *P* = 0.004). Multiple vessels stenosis and multifocal concentric VW enhancement were statistically more frequent in the ACNS group (71 vs. 35%, *P* = 0.03 and 95 vs. 4%, *P* = 0.004, respectively). On the contrary, eccentric VW abnormalities were seen exclusively in the stroke-unit-group. Two patients in the ACNS group did not presented a typical IVW enhancement, while a multifocal concentric IVW enhancement was described in two patients in the stroke-unit-group for whom a diagnosis of ACNS could not be retained. Thus, multifocal concentric VW enhancement held a specificity of 95% and a sensitivity of 94% for ACNS diagnosis in our cohort (Table 3).

**Table 3:** Details about specificity and sensitivity computation for multifocal concentric vessel enhancement to diagnose ACNS.

		**ACNS**		**TOT**
		**No**	**Yes**	
**MF CONC IVW**	No	46	2	48
	Yes	2	36	38
**TOT**		48	38	86

*MF CONC IVW, multifocal concentric vessel enhancement in intracranial vessel wall imaging; ACNS, angiitis of the central nervous system, TOT, total*.

*Sensitivity: 36 (true positive)/[36+2(false negative)] = 94%.*

*Specificity: 46 (true negative)/[46+2(false positive)] = 95%*.

## Discussion

This work shows that IVW imaging IVW displays a 95% specificity and 94% sensitivity for the diagnosis of brain vasculitis.

The characteristics of our patients are comparable with those of previously reported ACNS cohorts, in terms of acute symptoms onset and cognitive deficit, percentage of abnormal CSF samples, white-matter hyperintense FLAIR lesions and haemorrhagic manifestations at MRI suggesting that our results can be generalizable to other cohorts of ACNS. It has to be noted, however, that concerning the percentage of patients presenting with seizure, the proportion of acute ischemic lesion on MRI, of vascular abnormalities (multifocal stenosis and VW enhancement) on MRA and IVW-MRI and the range of the elevation of white blood cells counts and protein levels in the CSF, our cohort are more in line with DSA-diagnosed vasculitis than biopsy-diagnosed ones (de Boysson et al., 2014; Salvarani et al., 2015b; Schuster et al., 2017). These findings are not surprising. Since almost the whole of our cohort presented IVW vessels imaging abnormalities considered typical for ACNS (95%), it probably mainly consisted of patients with medium-sized vessel vasculitis. This subtype of vasculitis is typically diagnosed by DSA and biopsy in these patients may be normal (Schuster et al., 2017). On the contrary, ACNS affecting smaller vessels are usually diagnosed by biopsy and DSA might be negative because the diameter of the affected vessels is beyond the resolution of vascular imaging (Schuster et al., 2017). Compared to other cohorts, headache was less frequent in our population [29 vs. 59% (Salvarani et al., 2015b) or 52% (Boulouis et al., 2017)]. This finding is probably due to a selection bias since we considered for inclusion only patients admitted in the stroke unit. The stroke recruitment bias probably explains also the difference in the prevalence of focal neurological deficit which were constant in our cohort whereas they were present in 80–85% of cases in previous series (de Boysson et al., 2014; Boulouis et al., 2017). The patients in our cohort were older than in previously reported studies (as an example 60 (range [17–84]) years in our cohort vs. 43 (range [18–79]) years in the COVAC cohort). Since in our institution the imaging protocol frequently includes IVW sequences, we have probably more deeply investigated patients that would have normally not benefited of a complete ACNS work-up otherwise. Despite these differences due to a selection bias, our findings suggest that our cohort corresponds to previously described DSA-diagnosed vasculitis patients and that IVW imaging might be a valid alternative to more invasive procedures for diagnostic work-up in case of ACNS suspicion. Further study are necessary to define if an abnormal IVW may waive the need for DSA for probable vasculitis diagnosis.

ACNS diagnosis is challenging and IVW imaging will probably play a significant role in the future thanks to their specificity and safeness. In our cohort, IVW imaging holds a specificity of about 95% and a sensitivity of 94% for ACNS diagnosis. Concerning specificity, it has to be noted that in one out of two patients in the stroke-unit-group, who presented with a concentric and multifocal VW enhancement, a diagnosis of ACNS may not be retained because of an incomplete work-up. Furthermore, the ACNS and the stroke-unit-group were superimposable in terms of age, gender balance, clinical symptoms (except for cognitive disorders) and cardiovascular risk factors, therefore the differences in the IVW results should not have been influenced by a selection bias. Among vascular imaging, DSA has been considered as the gold standard for ACNS diagnosis. In our cohort, angiographic abnormalities were associated with concentric vessel wall enhancement, results in agreement with previously reported series (Van Rooij et al., 2018; Thaler et al., 2019) that confirm the validity of the VW technic. However, DSA is invasive, it holds a variable sensitivity (from 27 to 90%) and its specificity can be as low as 30% as other intracranial vasculopathies than ACNS may present a similar pattern of multiple stenosis in different arterial territories (Duna and Calabrese, 1995; Alexander et al., 2016; Singhal et al., 2016). Previous works have employed MRA in the ACNS work-up to remedy to the invasiveness of DSA. In various studies derived from the French cohort COVAC, authors showed that TOF-MRA could identify the 90% of vessels abnormalities found in DSA positive ACNS patients (Boulouis et al., 2017; de Boysson et al., 2018). Moreover, applying higher field strengths, TOF-MRA detection rates of stenosis can be increased (Cosottini et al., 2013; de Boysson et al., 2017), to the point that concordance between 3D-TOF-MRA and DSA for the identification of arterial stenosis in ACNS patients is 0.87 (95% CI: 0.78–0.91) (de Boysson et al., 2017). They conclude that, in case of negative 3T 3D-TOF-MRA findings, the added diagnostic value of DSA is limited (de Boysson et al., 2017). However, the question whether TOF-MRA may replace DSA is still a matter of debate. In our cohort, abnormalities in IVW imaging were found in five patients with normal TOF-MRA sequences and such a finding confirms the sensitivity gain of VW techniques, in accordance to previously reported data (Patzig et al., 2022). In fact, the main limitation of conventional vascular technics is that they can identify only abnormalities affecting the vessel lumen which may be the result of different pathophysiological processes. On the contrary, IVW can directly characterize disease processes affecting the walls of arteries providing diagnostic information not available with traditional angiographic techniques (Alexander et al., 2016). IVW abnormalities in ACNS are characterized by a multifocal or diffuse homogenous concentric enhancement of the vessel wall that it usually thickened (Kanduumlker et al., 2008; Obusez et al., 2014). Vessel wall contrast enhancement is presumed to be caused by hyperpermeability of the endothelium and/or by neovascularization, resulting in leakage of contrast into the arterial wall (Mandell et al., 2017). The inflammatory involvement of arterial segments showing VW enhancement on MRI was histologically confirmed in some reports (Van Rooij et al., 2018). Finally, information derived from this technic might become useful for prognostication too. In a study on ACNS patients, those presenting with a greater number of affected vessels had a greater chance to escape treatment and relapse (Yang et al., 2021). Furthermore, we showed that the progression of IVW abnormalities in follow-up MRI was associated with a higher prevalence of relapses.

IVW can also significantly improve the differentiation of intracranial vasculopathies, particularly when combined with traditional luminal imaging modalities (Mossa-Basha et al., 2017). In ICAD, for example, the vessel wall enhancement is typically eccentric (Mossa-Basha et al., 2015; Alexander et al., 2016) as it was reported in 8 patients in our stroke-unit-group. Moreover, IVW studies provided some prognostic information about ICAD stroke patients showing that higher plaque enhancement is associated with a higher probability of recurrent stroke (Sun et al., 2021). Since ICAD is a common cause of ischemic stroke (Hurford et al., 2020), the risk for intracranial stenosis increase with age (form <10% in <50 years to > 45% in > 90 years) (Hurford et al., 2020) and stroke recurrence remains high despite aggressive medical management (Turan et al., 2017), IVW should be routinely used in stroke patients to improve diagnostic sensibility and provide a more patient-oriented treatment.

There are, however, several caveats for the employment of IVW technics in ACNS diagnostic. Different patterns of vessels abnormalities have been recognized in ACNS patients that, although less frequent than the homogenous concentric thickening and enhancement of the vessel walls, might complicate the differential diagnosis (Obusez et al., 2014). Furthermore, some other pathological conditions like ICAD, RCVS and Moyamoya disease may present with similar IVW abnormalities (Kim et al., 2013; Ryoo et al., 2014; Alexander et al., 2016). Also, there might be a discrepancy between the severity of intracranial vessel walls involvement on IVW-MR and the clinical disease activity (Mandell et al., 2017). As showed in our cohort and in accordance with the literature (Patzig et al., 2022), IVW abnormalities may persist despite treatment in clinically improving patients making risky to lay on IVW sequences for monitoring response to therapy (Patzig et al., 2022). Finally, data in the literature reported significant less frequent IVW abnormalities in small, i.e., biopsy-positive DSA-negative ACNS, than in medium-large vessel angiitis (Thaler et al., 2019; Patzig et al., 2022). Nevertheless, few case of patients with a biopsy-driven diagnosis of ACNS without anomalies in intraluminal vessel imaging (MRA or DSA) but presenting with typical IVW abnormalities can be found in published studies (Kanduumlker et al., 2008; Patzig et al., 2022). These findings suggest that, probably due to the heterogeneity of the spectrum of inflammatory vasculopathies (Patzig et al., 2022), IVW might be less useful in small-vessel-size DSA-negative ACNS diagnosis.

Our study presents several limitations. According to the diagnostic criteria for CNS vasculitis, firstly proposed in 1988 (Calabrese and Mallek, 1988) and updated in 2009 (Birnbaum and Hellmann, 2009), the diagnosis is considered as *definite* only in case of consistent tissue biopsy. Otherwise, a *probable* diagnosis maybe retained if high-probability findings on angiogram are supported by abnormal parenchymal MRI and CSF samples. Since, in the majority of our cohort, DSA was not performed, the diagnosis does not always fulfill those criteria. Nevertheless, those criteria highlighted the importance to base diagnosis on a beam of arguments (intraluminal and vessel wall imaging, parenchymal abnormalities, CSF analysis) namely to discriminate between RVCS and vasculitis. Since none of our patients presented a clinical picture compatible with RCVS and that ACNS diagnosis was based on several abnormal ancillary exams, we deem to have included patients with *probable* ACNS. However, since clinicians were not blinded to IVW results, we cannot exclude that those MRI information may have partially influenced clinical judgment for inclusion. The largest described cohort of ACNS, that we considered for comparison, included only patients with PACNS (Salvarani et al., 2015b; Boulouis et al., 2017). Despite they may present some pathophysiological differences, primary and secondary ACNS share many clinical and iconographic features (Scolding, 2009), namely IWV sequences, so we decided to consider them together. As discussed above, the high prevalence of IVW abnormalities and low prevalence of biopsy-positive/DSA-negative patients reflect the fact that we probably included an higher percentage of medium-sized vessels vasculitis subtype than reported in previous cohorts (de Boysson et al., 2014; Salvarani et al., 2015b). Therefore, our results about IVW sensitivity and specificity may not apply to the whole population of ACNS. The inclusion, in the stroke-unit group, of patients without cerebral vessel stenosis, despite probably more clinically relevant, might have overestimate the specificity of the IVW. We performed the statistical analysis considering only patients in the control group presenting with cerebral vessel stenosis and the results confirmed the good capacity of multifocal concentric IVW enhancement to discriminate ACNS from other causes of cerebral vessel stenosis (specificity slightly decreased from 95 to 90%, sensitivity remained stable at 94%).

Finally, the sample size probably limited the significance of certain statistical analysis. Our study provides an additional cue to promote IVW sequences employment in clinical practice. Nevertheless, further studies analyzing IVW abnormalities in patients with ACNS diagnosed according to the current guidelines, are necessary to quantify the diagnostic value of IVW sequences and to establish if they can replace a standardized diagnostic approach.

### Conclusions

Our results show that the concentric VW enhancement can efficaciously identify patients affected by medium-sized vessels CNS vasculitis with a specificity of 95% and sensitivity of 94%. Furthermore, IVW sequences can help in the differential diagnosis of intracranial vasculopathies, providing useful information about likely disease evolution and should be included in the standard work-up of patients presenting with acute neurologic deficit and stroke.

## Data availability statement

The datasets used and/or analyzed during the current study are available from the corresponding author on reasonable request.

## Ethics statement

The studies involving human participants were reviewed and approved by Ethics Committee of Erasme Hospital (reference P2020/736). Written informed consent for participation was not required for this study in accordance with the national legislation and the institutional requirements.

## Author contributions

LF, NL, AR, and VD organized the database and performed the statistical analysis. LF wrote the first draft of the manuscript under the direct supervision of GN and NL. NS acquired and interpreted the neuroimaging data. GN and NL conceived and designed the study. All authors contributed to manuscript revision, read, and approved the submitted version.

## Conflict of interest

The authors declare that the research was conducted in the absence of any commercial or financial relationships that could be construed as a potential conflict of interest.

## Publisher's note

All claims expressed in this article are solely those of the authors and do not necessarily represent those of their affiliated organizations, or those of the publisher, the editors and the reviewers. Any product that may be evaluated in this article, or claim that may be made by its manufacturer, is not guaranteed or endorsed by the publisher.
